# Squamous cell lung carcinoma in a patient with epidermodysplasia verruciformis

**DOI:** 10.1016/j.jdcr.2024.01.030

**Published:** 2024-02-14

**Authors:** Jason Eakes, Meredith Thomley, Nirav Shah, Sebastian Verne, Jane Messina, Tadeu Ambros, Lilia Correa-Selm

**Affiliations:** a USF Health Morsani College of Medicine, University of South Florida, Tampa, Florida; b Department of Dermatology and Cutaneous Surgery, USF Health Morsani College of Medicine, University of South Florida, Tampa, Florida; c Department of Pathology and Cell Biology, USF Health Morsani College of Medicine, University of South Florida, Tampa, Florida; d Department of Cutaneous Oncology, H. Lee Moffitt Cancer Center & Research Institute, Tampa, Florida; e Department of Pathology, H. Lee Moffitt Cancer Center & Research Institute, Tampa, Florida; f Florida Cancer Specialists & Research Institute, Fort Myers, Florida

**Keywords:** epidermodysplasia verruciformis, EV, HPV, human papillomavirus, lung cancer, SCC, skin cancer, squamous cell carcinoma, squamous cell lung carcinoma

## Introduction

Epidermodysplasia verruciformis (EV) is a rare autosomal recessive disorder characterized by increased susceptibility to genus β human papillomavirus (HPV) skin infections.^1^ HPV types 5, 8, 9, 12, 14, 15, 17, and 19-25 are the most commonly associated types present in EV patients.^2^ Most concerning of these include HPV types 5 and 8 which have been implicated for their carcinogenic potential in this population.^1^, ^2^, ^3^ The most common genetic mutations responsible, *TMC6*/EVER1 and *TMC8*/EVER2, are part of an immune complex that prevent HPV replication. Therefore, mutations in either of these proteins result in unregulated viral replication and ultimately persistence of HPV infection.^4^ Clinically, this presents as widespread papules and plaques resembling flat warts, generalized erythematous macules and plaques resembling pityriasis versicolor, or seborrheic keratosis-like plaques.^1^^,^^3^ The resulting lesions are pervasive and have potential to transform into cutaneous squamous cell carcinoma (SCC), with over one-half of patients developing skin cancer by the fourth-fifth decade of life.^1^, ^2^, ^3^, ^4^, ^5^ Cutaneous SCC in EV can arise in any location on the body but favors sun exposed areas, as UV exposure confers malignant transformation in these genetically susceptible patients.^1^, ^2^, ^3^, ^4^ While EV is known to increase the risk of skin cancer, development of internal malignancy is very unusual, with 1 case reported in the literature.^6^ Here, we present a case of primary squamous cell lung carcinoma in a young patient with EV and speculate on the role her heritable, HPV-driven disorder may have played in leading to internal malignancy.

## Case report

**Fig 1:**
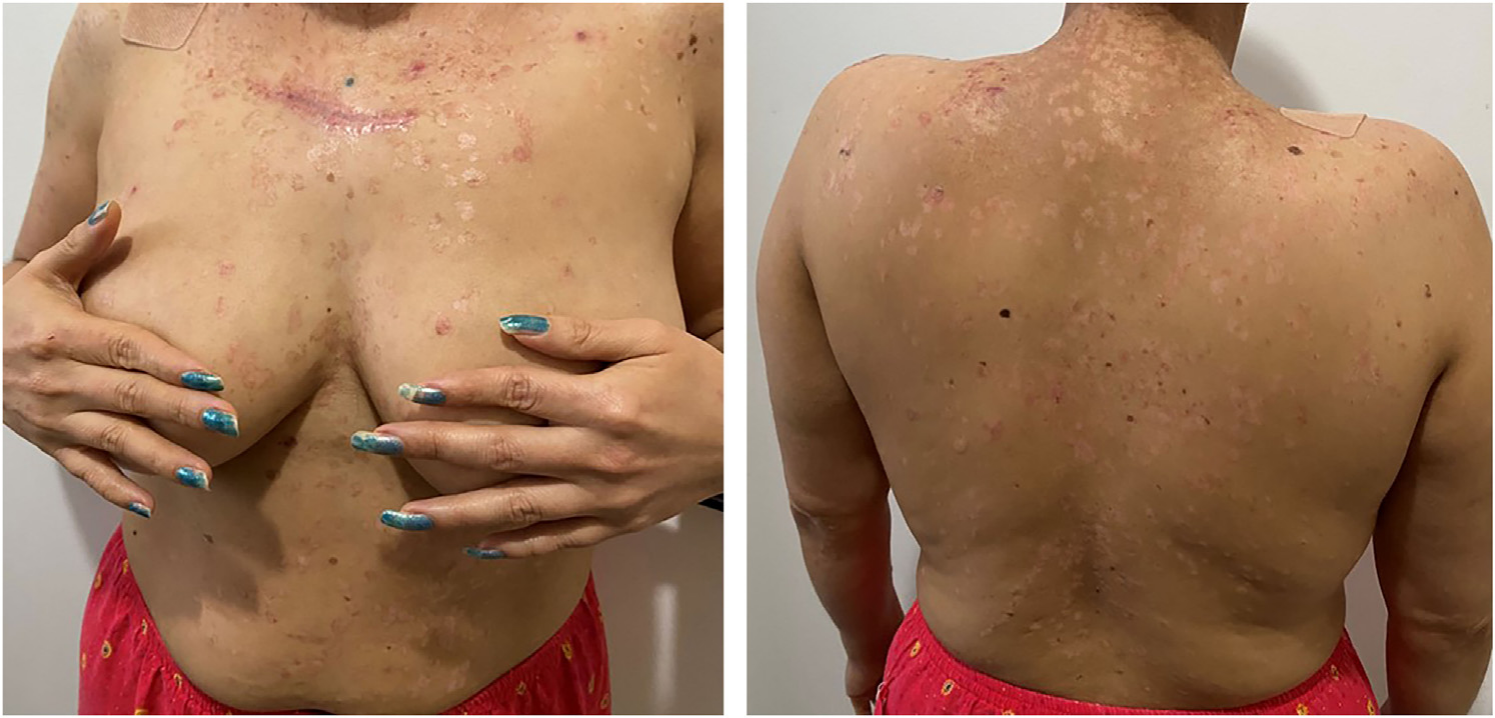
Epidermodysplasia verruciformis.

**Fig 2:**
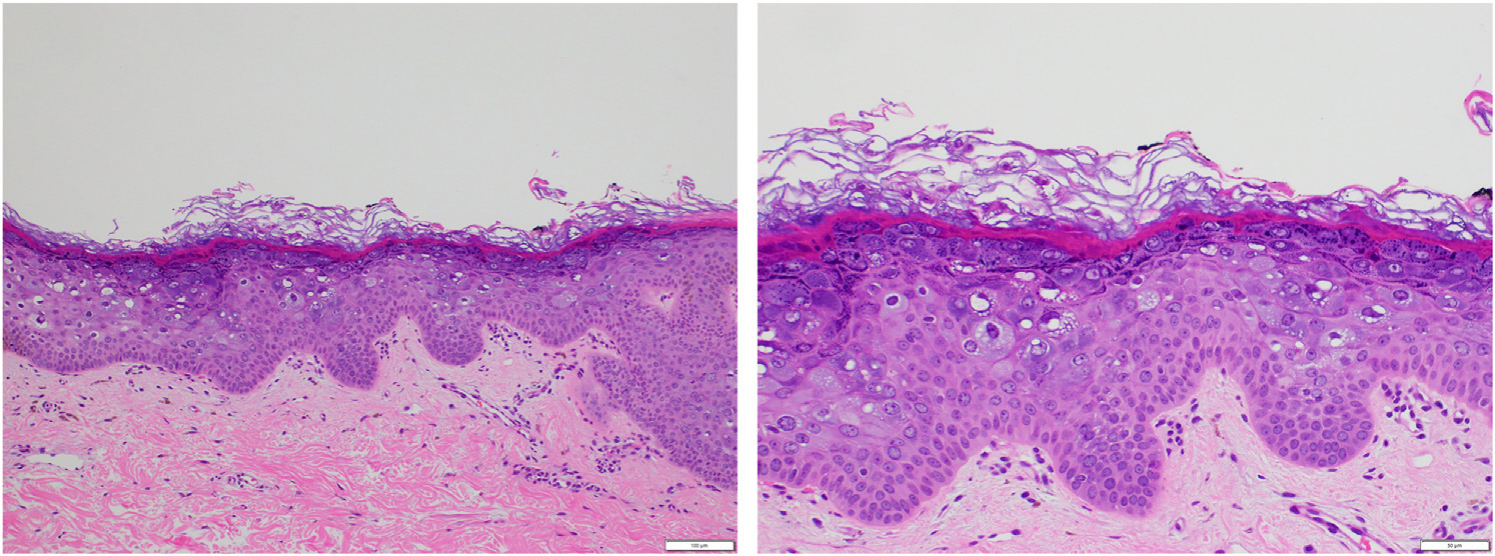
Shave biopsy with hematoxylin and eosin stain.

A 38-year-old female presents to our clinic with a history of numerous cutaneous SCCs in the past decade. The patient was born in El Salvador and reports a history of rash starting at age three. Since childhood, she gradually developed multiple tan-pink macules and flat-topped papules, most prominently on the face, trunk, and extremities which were largely asymptomatic (Figs 1 and 2). She sought care in her birth country for this, as well as the development of 5+ SCCs of the skin including 1 that metastasized to her right orbit and neck. She presented to our cancer center for follow-up on her history of skin cancers, which had been previously treated with a variety of modalities including chemotherapy, radiation, excisions, and cryotherapy in El Salvador. Eight months prior, the patient was also diagnosed with primary squamous cell lung carcinoma and was treated with carboplatin, pembrolizumab, and paclitaxel with initial good response. Given the patient’s age and development of numerous SCCs, she underwent genetic testing for Xeroderma Pigmentosum and EV which revealed a mutation in the *TMC8* gene associated with EV. The physical exam was remarkable for numerous, 3-8 mm light tan-pink, round-to-oval, papules with confluence into plaques, some with fine scale, located throughout the trunk and extremities.

A shave biopsy of a right shoulder lesion revealed multiple large keratinocytes with blue-gray cytoplasm and varying-sized keratohyalin granules within the upper spinous and granular layers. Taken together, these findings favor a diagnosis of EV. Other considerations for differential diagnoses included disseminated superficial actinic porokeratosis, pityriasis versicolor, and acquired EV from secondary immunodeficiency such as HIV or lymphoma.

Imiquimod and photodynamic therapy were recommended for nonfacial and facial lesions, respectively, with continued pembrolizumab per oncology. Frequent skin exams, sun protective behaviors, genetic testing, niacinamide supplementation, and HPV vaccination were also encouraged. After 10 months of treatment, pembrolizumab was discontinued. However, after a courageous battle with lung cancer, the patient regrettably passed away several months later.

## Discussion

Patients with EV have unchecked HPV replication and persistence of infection.^4^ This persistence is partly due to the markedly reduced population of Langerhans cells in HPV-infected lesions resulting from reduced chemotactic signaling from infected keratinocytes.^2^ Additionally, the commonly mutated genes, *TMC6*/EVER1 and *TMC8*/EVER2, are highly-expressed in CD4^+^ and CD8^+^ T lymphocytes, B lymphocytes, and NK cells and cause a dysfunctional response in EV.^5^ Therefore, patients with EV are widely predisposed to the development of skin cancer through unchecked HPV infection. However, development of internal malignancy has only been demonstrated once.^6^ In 2011, Rottenberg et al reported development of poorly differentiated primary SCC of the lung in 21-year-old patient with a history of cutaneous SCC and EV. Further cytological analysis of the malignant cells favored a diagnosis of new primary lung SCC over secondary metastasis from skin SCC to lung.^6^ The mechanism for this development likely revolves around altered adaptive immune function. Recently, it was shown that increased expression of *TMC8* in lymphocytes predicted improved prognoses in head and neck SCC primarily through increased activation and infiltration of CD4^+^ T cells, CD8^+^ T cells, B cells, and macrophages.^7^ Further, expression of *TMC8* was significantly upregulated in patients with cancers that were HPV-positive compared to HPV-negative implying its significance in defense against HPV-derived cancers.^7^ The role of cellular immunity has also been implicated in the accelerated development of cutaneous SCC in cases of atypical EV during immunosuppressive states such as organ tranplantation.^3^ Furthermore, as recently demonstrated, infection with HPV might increase the risk of developing lung cancer even in immunocompetent patients without diagnosis of EV.^8^ Taken together, the findings of primary SCC in a young patient without other identifiable risk factors, the role of cellular immunity in EV, and the potential dual increased risk of lung cancer from HPV-infection and impaired adaptive immunity support a positive association between EV and the subsequent development of primary lung SCC in this patient. Subsequent investigations must explore this relationship and provide guidance on novel therapeutics.

## Conflicts of interest

Dr Correa is a consultant for Accutec, a consultant and researcher for Novartis Pharmaceutical and a researcher for Pfizer. She also serves on the Advisory Board for the Jacinto Convit World Organization and the Dermatology Advisory for Melanoma Research Foundation. All other authors declare no conflicts of interest.
